# Society Meetings

**Published:** 1916-07

**Authors:** 


### SOCIETY MEETINGS

---

**Brief reports and announcements of meetings of societies of Western New York, and those of general scope, are requested from Secretaries. Copy should be on hand the fifteenth of the month. Full transactions will be published at cost of composition.**

---

**DOCTOR: Is your Society properly represented here? If not, please bring our standing announcement to the attention of the members.**

---

**The American Medical Editors' Association** will meet at the McAlpine Hotel, New York, October 25th and 26th.

The Local Committee comprises: Dr. Thomas L. Stedman, (Editor, Med'l Record) Chairman; Dr. R. H. Sayre, (New York Medical Journal); Dr. Brooks H. Wells, (Editor, Amer. Jr'l of Obstetrics); Dr. Frank C. Lewis, (Internat'l Jr'l of Surgery); Dr. Ira S. Wile, (American Medicine).

The officers of the Association for 1915 and 1916 are as follows: Dr. Edward C. Register, President, (Charlotte

Med'l Journal, Charlotte, N. C.); Dr. W. A. Jones, 1st Vice-President, (Journal Lancet, Minneapolis, Minn.); Dr. G. M. Piersol, 2nd Vice-President, (Amer. Jr'l Med'l Sciences, Philadelphia, Pa.); Dr. J. McDonald, Jr., Secretary and Treasurer, (American Journal of Surgery, New York.)

Executive Committee: Dr. C. F. Taylor, (Medical World, Philadelphia, Pa.); Dr. John C. MacEvitt, (N. Y. State Jr'l of Medicine, New York); Dr. A. S. Burdick, (Amer. Jr'l of Clin. Med., Chicago, Ill.); Dr. Joseph MacDonald, Jr., (Amer. Jr'l of Surgery, New York).

The meeting on October 25th and 26th, will be devoted exclusively to problems of strictly journalistic nature, which will be of importance and interest to every editor and publisher of a medical journal. Among the papers to be presented are the following:

"Editorial Control"—"The Editor's Prerogative in Editing Original Articles"—"Book Reviews in Medical Journal"—"Problems of the Subscription Department"—"The Relationship Between Medical Journals of the Day"—"The Up-Lift in Medical Journalism"—"The Influence of the Medical Press and Profession in Public Affairs"—"The Rights of an Author in the Disposition of his Contribution," etc.

---

**The Alumni Association of the Medical Dept. of the University of Buffalo** will publish its proceedings in the August issue.

---

**The A. M. A. Meeting** at Detroit, was one of the largest ever held, the registration of physicians being nearly 4500. The maximum attendance was at Chicago, 1908, 7000 and the next at Boston, 1906, 6000. Allowing for obvious differences in the potential attendance from the city of meeting and the immediate vicinity, the Detroit meeting may be considered the most successful to date. The arrangements were thoroughly made and comfort and convenience were attained to a higher degree than is possible for most meeting places outside of Atlantic City. The hospitality was liberal and cordial. One of the most practical features was the volunteering of automobiles by physicians and others for the use of guests. A conspicious A. M. A. monogram on the wind shield indicated that the car was at the service of any guest who hailed it. Messrs. Parke Davis & Co. kept an automobile service running between Headquarters and their plant, conducting visitors through the scientific laboratories and other parts of their manufactory and dispensed refreshments in a markee. The Ford and other automobile plants also attracted many guests. The newspapers gently jested the medical profession on its lack of attention to ventilation of its meeting places and on the use of high-heeled shoes by the great majority of women physicians and doctors' wives. As the former criticism was beyond the control of either professional hosts or guests and as the latter was considered ultra vires, so far as medical men are concerned, some good may be expected from them, especially as the press was most courteous in its news service. The next meeting of the A. M. A. will be held in New York, under the presidency of Dr. Wm. H. Mayo of Rochester, Minn., Surgeon General Rupert Blue of the U. S. Public Health Service then retiring.

---

**The Western N. Y. Veterinary Medical Assn.** held its annual meeting in Buffalo in June, Dr. E. L. Volgenau of Buffalo, Pres.; Dr. J. L. Wilder of Akron, Vice-Pres.; Dr. F. F. Fehr of Buffalo, Sec.-Treas.

---

**The Dental Society of New York State** held its annual meeting at Albany in May. Officers were elected as follows: Dr. Robert Murray of Buffalo, Prs.; Dr. Amos C. Rich of Saratoga Springs, V. P.; Dr. Geo. H. Butler of Syracuse, Treas.; Dr. A. P. Burkhart of Auburn, Sec.; Dr. I. L. M. Waugh of N. Y. (formerly of Buffalo) Corresponding Sec.

---

**The Elmira Academy of Medicine Held a Regular Meeting** June 7. Dr. Palmer H. Lyon of Valois, gave a paper on Rational Methods in Treatment of Neurasthenia; Dr. Anna Stuart of Elmira, on Infantile Scurvy.

---

**The Buffalo Academy of Medicine** has held the following meetings since our last report: Section of Obstetrics and Gynaecology, May 17, Extra-uterine Pregnancy Aetiology, Dr. C. A. Bentz; Diagnosis and Treatment, Dr. Earl P. Lothrop.

Section of Pathology, May 24, Preventable Deafness, Dr. Clayton M. Brown; Mastoiditis, Diagnosis and Treatment, Dr. John F. Fairbairn.

Annual Meeting and Election of Academy, June 14.

The Officers for the following year are as follows: President, Dr. Lawrence Hendee; Secretary, Dr. Herbert A. Smith; Treasurer, Dr. Bernard F. Schreiner; Trustee, Dr. Wm. Ward Plummer. The Chairmen and Secretaries of Sections: Surgery, Dr. F. M. O'Gorman and Dr. James Cross; Medicine, Dr. E. A. Sharp and Dr. Harry Mead; Obstetrics,

Dr. Irving M. Potter and Herman K. De Groat; Pathology, Dr. Wm. F. Jacobs and Dr. Wm. G. Bissell.

---

**Medical Society, County of Erie.** The regular meeting of this Society was held at the University of Buffalo, No. 24 High Street, on June 19th, 1916, at 8:45 p. m., Dr. Franklin W. Barrows, President, presiding.

The Secretary read the minutes of the last regular meeting held April 17th, 1916, also of the joint meeting of the Council and the Delegates to the State Society held on May 11th, 1916, also the minutes of the Council held June 6th, 1916, all of which were approved as read.

In the absence of Dr. Jacobs, Chairman of the Committee on Membership, Dr. Jesse N. Roe of the Membership Committee presented the names of the following applicants and on behalf of the Committee recommended their election. These names had been previously presented to and favorably acted upon by the Council.

Dr. Leo. A. Bussman, 690 Broadway, Buffalo, a graduate of the University of Buffalo of 1913.

Dr. Francis W. Leopold, 36 Woltz Avenue, a graduate of the University of Buffalo, 1914.

Reinstatement of Dr. George Marshall Lewis, 753 Amherst Street, Buffalo.

Reinstatement of Dr. Sydney A. Dunham, 1392 Amherst Street, Buffalo.

Reinstatement of Dr. Hugh J. McGee, 820 Elk Street, Buffalo.

Reinstatement of Dr. William Hoddick, 68 Barker Street.

Reinstatement of Dr. Clarence A. Tyler, Alden, N. Y.

Each one of these applicants were separately balloted upon and elected and reinstated.

Treasurer Lytle stated that at the last meeting of the Society he was excused by vote of those present from reading the names of members who were in arrears with their dues until a future meeting of the Society and asked the pleasure of the meeting.

On motion of Dr. Bonnar the reading of the names of members in arrears were suspended.

Dr. Lytle then read the resignation of Dr. Leonard Reu, which he had just received and moved that it be referred to the Committee on Membership. Carried.

Dr. Bonnar made a brief report of the Board of Censors giving an account of the activities of the Board since last meeting of the Society, and what had been done and accomplished with offenders of the medical practice law.

This concluded the business session and the scientific program consisted of a clinic in which a number of patients were presented by Dr. George F. Cott. The clinical history and condition of each patient was carefully gone over by Dr. Cott and proved to be very interesting to the Society. Following the clinic Dr. Cott gave a lantern slide demonstration.

Dr. James A. Gibson presented a very interesting series of lantern slides which showed the Pituitary Body, showing its normal condition and also the many deviations from the normal as well as the results following such abnormals.

At the conclusion of these demonstrations the subject matter presented was discussed by the Society.

Dr. C. R. Borzilleri presented some pathological specimens and gave the history of the case.

The Society then adjourned.

